# Transplacental Transmission of SARS-CoV-2: A Narrative Review

**DOI:** 10.3390/medicina60091517

**Published:** 2024-09-18

**Authors:** Minh Tien Bui, Cam Anh Nguyen Le, Khanh Linh Duong, Van Thuan Hoang, Trung Kien Nguyen

**Affiliations:** Thai Binh University of Medicine and Pharmacy, Thai Binh 410000, Vietnam; tienbm@tbmc.edu.vn (M.T.B.); nguyencamanh3@gmail.com (C.A.N.L.); linh.duongkhanh178@gmail.com (K.L.D.); thuanytb36c@gmail.com (V.T.H.)

**Keywords:** transplacental transmission, SARS-CoV-2, COVID-19

## Abstract

*Background and Objectives:* The study aims to explore the potential for transplacental transmission of SARS-CoV-2, focusing on its pathophysiology, placental defense mechanisms, and the clinical implications for maternal and neonatal health. *Materials and Methods:* A comprehensive review of the current literature was conducted, analyzing studies on SARS-CoV-2 infection in pregnancy, the expression of key viral receptors (ACE2 and TMPRSS2) in placental cells, and the immune responses involved in placental defense. The review also examined the clinical outcomes related to maternal and neonatal health, including adverse pregnancy outcomes and neonatal infection. *Results:* The expression of ACE2 and TMPRSS2 in the placenta supports the biological plausibility of SARS-CoV-2 transplacental transmission. Histopathological findings from the infected placentas reveal inflammation, vascular changes, and the evidence of viral particles in placental tissues. Clinical reports indicate an increased risk of preterm birth, intrauterine growth restriction, and neonatal infection in pregnancies affected by COVID-19. However, the frequency and mechanisms of vertical transmission remain variable across studies, highlighting the need for standardized research protocols. *Conclusions:* SARS-CoV-2 can potentially infect placental cells, leading to adverse pregnancy outcomes and neonatal infection. While evidence of transplacental transmission has been documented, the risk and mechanisms are not fully understood. Ongoing research is essential to clarify these aspects and inform obstetric care practices to improve maternal and neonatal outcomes during the COVID-19 pandemic.

## 1. Introduction

The emergence of the severe acute respiratory syndrome coronavirus 2 (SARS-CoV-2) and the subsequent COVID-19 pandemic have posed unprecedented challenges to global public health. This novel coronavirus, first identified in December 2019 in Wuhan, China, rapidly spread worldwide, leading to widespread morbidity and mortality. While extensive research has focused on understanding the transmission dynamics, clinical manifestations, and management of COVID-19, specific concerns have arisen regarding the impact of the virus on pregnant women and their fetuses [1].

Pregnancy is a unique physiological state characterized by significant alterations in the immune, cardiovascular, and respiratory systems [2]. These changes can render pregnant women more susceptible to infections and severe disease outcomes. Historically, other coronaviruses, such as those causing severe acute respiratory syndrome (SARS) and Middle East respiratory syndrome (MERS), have been associated with adverse pregnancy outcomes, including miscarriage, preterm birth, and maternal mortality [3]. Consequently, there has been considerable interest in investigating the effects of SARS-CoV-2 on pregnancy and the potential for vertical transmission [4].

Vertical transmission, or mother-to-child transmission, can occur through various routes, including during pregnancy (transplacental), delivery (intrapartum), or after birth (postpartum). Transplacental transmission specifically refers to the passage of pathogens from the mother to the fetus via the placenta [5]. The placenta acts as a critical barrier, protecting the fetus from various infections while allowing the exchange of nutrients and waste products. However, certain pathogens can breach this barrier, leading to fetal infection and potential adverse outcomes [6].

The possibility of transplacental transmission of SARS-CoV-2 has been a subject of intense scrutiny. Early in the pandemic, reports of newborns testing positive for SARS-CoV-2 shortly after birth raised concerns about the virus’ ability to cross the placental barrier [7,8,9,10]. Subsequent studies have sought to elucidate the mechanisms by which SARS-CoV-2 might infect placental cells and the implications of such infections for maternal and fetal health [11,12,13,14,15,16,17].

In addition to SARS-CoV-2, the impact of other respiratory viruses during pregnancy provides valuable context for understanding the implications of viral infections on maternal and fetal health [18,19,20]. Influenza virus, for example, is a well-documented respiratory pathogen that poses significant risks to pregnant women and their offspring [21,22,23]. Pregnant women are at increased risk of severe influenza-related complications due to physiological changes that affect immune response and respiratory function [21,24]. Studies have shown that influenza infection during pregnancy can lead to adverse outcomes such as preterm birth, low birth weight, and increased risk of neonatal hospitalization [23,25,26]. Furthermore, the respiratory syncytial virus (RSV) has been associated with heightened morbidity and mortality in infants when transmitted from mothers who are infected during pregnancy [27,28]. By examining these infections, it becomes evident that viral respiratory illnesses can significantly affect pregnancy outcomes and neonatal health. Such comparisons underscore the importance of understanding SARS-CoV-2’s transplacental transmission and its potential consequences, thereby providing a more comprehensive perspective on the impact of respiratory viruses during pregnancy.

## 2. Pathophysiology of Transplacental Transmission

Understanding the pathophysiology of transplacental transmission of SARS-CoV-2 involves examining the structure and function of the placenta as well as the molecular mechanisms by which the virus can infect placental cells [29]. The primary receptor for SARS-CoV-2, angiotensin-converting enzyme 2 (ACE2), is expressed in various placental cell types, including trophoblasts and syncytiotrophoblasts. These cells are critical components of the placental barrier and play essential roles in nutrient exchange and hormone production [30,31].

The entry of SARS-CoV-2 into host cells is facilitated by the spike (S) protein, which binds to ACE2 and is subsequently cleaved by the transmembrane serine protease 2 (TMPRSS2). The presence of ACE2 and TMPRSS2 in the placenta supports the biological plausibility of SARS-CoV-2 infection of placental cells [32]. Once the virus infects these cells, it can potentially cause placental inflammation, vascular damage, and thrombotic events, which may impact fetal health [33].

Transplacental transmission of SARS-CoV-2 involves complex interactions between the virus and the maternal–fetal interface [34,35]. The placenta, a multifaceted organ, not only facilitates nutrient and waste exchange but also serves as a critical barrier against pathogens. Understanding the mechanisms by which SARS-CoV-2 might breach this barrier and infect the fetus involves exploring the cellular and molecular dynamics of the placenta [33,36,37].

## 3. Role of Placental Structure and Function in SARS-CoV-2 Transplacental Transmission

The placenta is composed of several layers, each with distinct cellular components and functions. The outermost layer, the syncytiotrophoblast, is in direct contact with maternal blood and is primarily responsible for gas exchange, nutrient uptake, and waste removal [31]. Beneath this layer, the cytotrophoblasts serve as progenitor cells, continually fusing to replenish the syncytiotrophoblast. The underlying stromal layer contains blood vessels, immune cells, and connective tissue, providing structural support and mediating immune responses [38,39,40,41].

### 3.1. Angiotensin-Converting Enzyme 2

The primary receptor for SARS-CoV-2, angiotensin-converting enzyme 2 (ACE2), is expressed in various placental cell types, including trophoblasts and syncytiotrophoblasts. These cells are integral to the placental barrier and play essential roles in nutrient exchange and hormone production [30]. The expression of ACE2 in the placenta is dynamic, varying with gestational age and maternal health conditions. Higher levels of ACE2 have been observed in early pregnancy compared to later stages, potentially influencing the susceptibility of the placenta to viral infections at different pregnancy stages. Additionally, factors such as maternal hypertension and diabetes can modulate ACE2 expression, altering the risk of SARS-CoV-2 transmission [33].

### 3.2. Transmembrane Serine Protease 2

Transmembrane serine protease 2 (TMPRSS2) is another critical molecule involved in SARS-CoV-2 entry into host cells. The spike (S) protein of SARS-CoV-2 binds to ACE2 and is subsequently cleaved by TMPRSS2, facilitating viral entry and replication within placental cells. The presence of both ACE2 and TMPRSS2 in the placenta supports the biological plausibility of SARS-CoV-2 infection of placental cells [42].

### 3.3. Syncytiotrophoblast

The syncytiotrophoblast, while serving as a continuous barrier to pathogen entry, can be targeted by SARS-CoV-2 due to the presence of ACE2 receptors. Upon binding to ACE2, the S protein of the virus is cleaved by TMPRSS2, enabling the virus to enter and replicate within the cells. This process can lead to cellular damage, triggering inflammatory responses and compromising the placental barrier’s integrity [43].

Once inside the placenta, SARS-CoV-2 can elicit an immune response characterized by the release of pro-inflammatory cytokines and chemokines [44]. This inflammatory milieu can exacerbate placental damage, leading to conditions such as villitis (inflammation of the chorionic villi), deciduitis (inflammation of the decidua), and funisitis (inflammation of the umbilical cord) [45]. These conditions can impair placental function, reduce nutrient and oxygen delivery to the fetus, and increase the risk of adverse pregnancy outcomes, including preterm birth, growth restriction, and stillbirth.

Furthermore, SARS-CoV-2 infection can induce endothelial dysfunction and coagulopathy within the placenta, resulting in thrombosis and infarction. These vascular complications can compromise placental perfusion, further endangering fetal health. The pro-thrombotic state associated with COVID-19 in pregnant women can exacerbate these placental changes, highlighting the need for careful monitoring and management of affected pregnancies [46].

### 3.4. Alternative Entry Pathways

In addition to the well-characterized role of the angiotensin-converting enzyme 2 (ACE2) receptor and the serine protease TMPRSS2 in facilitating SARS-CoV-2 entry into host cells, emerging evidence suggests that other proteases may also play a role in the virus’ ability to infect placental cells [47]. Among these, cathepsin L and furin have garnered attention as potential contributors to viral entry, though their precise mechanisms and overall significance remain less well defined [48].

#### 3.4.1. Cathepsin L

Cathepsin L is a cysteine protease that is known to reside in lysosomes and endosomes, playing a critical role in protein degradation and processing. In the context of viral infections, cathepsin L can activate certain viruses by cleaving their spike proteins within endosomal compartments, facilitating membrane fusion and viral entry [49]. For SARS-CoV-2, studies have indicated that cathepsin L might serve as an alternative protease for spike protein priming, especially in scenarios where TMPRSS2 is less active or absent [50,51,52,53]. This suggests that in placental cells, cathepsin L could potentially aid in the virus’ ability to bypass the need for TMPRSS2, thereby enhancing its infectivity under certain conditions [54].

#### 3.4.2. Furin

Furin is a ubiquitously expressed proprotein convertase that cleaves and activates a variety of precursor proteins by recognizing a specific sequence motif [55]. The spike protein of SARS-CoV-2 contains a furin cleavage site, which is a distinctive feature that differentiates it from some other coronaviruses. This site allows furin to pre-activate the spike protein during viral assembly, increasing the efficiency of subsequent cell entry. In placental cells, furin’s presence could therefore provide an alternative route for SARS-CoV-2 to gain entry, independent of other proteases [56]. However, the extent to which furin contributes to placental cell infection in vivo is still under investigation.

## 4. Immune Responses and Placental Defense Mechanisms

The placenta is a critical organ in pregnancy, serving not only as a conduit for nutrients and gases between mother and fetus but also as a robust immunological barrier against infections. Its complex defense system includes multiple layers of innate and adaptive immune mechanisms that work in concert to protect the developing fetus from a wide range of pathogens, including viruses like SARS-CoV-2 [57].

### 4.1. Innate Immune Responses

One of the key components of placental immune defense is its ability to mount an innate immune response. The syncytiotrophoblast, which forms the outermost layer of the placenta, plays a central role in this process [58]. These cells, along with other placental cell types, can secrete a variety of cytokines, chemokines, and antiviral molecules in response to an infectious threat. These signaling molecules orchestrate a local immune response that can effectively limit the replication and spread of pathogens within the placenta. For instance, interferons produced by placental cells have been shown to have potent antiviral effects, enhancing the resistance of the placental barrier to viral invasion [59].

However, while the innate immune response is essential for controlling infections, it is a double-edged sword. The activation of immune pathways can also lead to inflammation, which may have detrimental effects on placental function and integrity [60]. In the context of SARS-CoV-2, heightened inflammatory responses have been associated with adverse pregnancy outcomes, such as preeclampsia and preterm birth, underscoring the delicate balance that must be maintained by the placental immune system [61,62].

### 4.2. Barrier Function

The physical barrier provided by the placenta is primarily attributed to the syncytiotrophoblast layer. This continuous, multinucleated layer of cells covers the entire surface of the placenta and is in direct contact with maternal blood. The syncytiotrophoblast is tightly sealed by intercellular tight junctions, which prevent the paracellular passage of pathogens. This barrier function is crucial in limiting the transplacental transmission of viruses like SARS-CoV-2 [43].

In addition to the syncytiotrophoblast, the placenta has other structural components, such as the basal lamina and the cytotrophoblast layer, that further reinforce its barrier capabilities. Together, these layers create a formidable obstacle that most pathogens find difficult to penetrate. However, certain factors, such as inflammation or damage to the syncytiotrophoblast, can compromise this barrier, potentially allowing pathogens to reach the fetal compartment [57].

### 4.3. Immune Cells

The placenta is also home to a diverse population of immune cells, including macrophages, dendritic cells, and natural killer (NK) cells, which are strategically positioned to detect and respond to pathogens. These immune cells contribute to the placenta’s defense through various mechanisms, including phagocytosis, antigen presentation, and the secretion of cytokines and chemokines [63].

Macrophages, known as Hofbauer cells in the placenta, are particularly important in managing infections. They can engulf pathogens, present antigens to other immune cells, and secrete factors that modulate the immune response. Dendritic cells, although less abundant, play a key role in initiating adaptive immune responses by presenting antigens to T cells, thereby linking innate and adaptive immunity [63].

The presence of these immune cells within the placenta not only helps in controlling infections but also in maintaining immune tolerance, ensuring that the maternal immune system does not mount an attack against the fetus, which is genetically distinct from the mother [64].

### 4.4. Gestational Age and Fetal Immune Response Factors in SARS-CoV-2

Gestational age appears to be a critical factor in the susceptibility and immune response of the fetus to SARS-CoV-2 infection. Studies have suggested that the immune system of the fetus evolves considerably during gestation, with significant differences in immune competence between early and late gestational periods [65]. In early gestation, the fetal immune system is relatively immature, relying predominantly on innate immunity, which may limit its ability to mount a robust response to viral pathogens, including SARS-CoV-2 [66]. As gestation progresses, particularly in the third trimester, the adaptive immune system begins to mature, with increasing numbers of functional T cells and the capacity for antibody production [67]. This enhanced immune capability may influence the fetal response to SARS-CoV-2 and impact the clinical outcomes of maternal infection [68]. However, it remains unclear whether this late gestation immune maturation confers protection against vertical transmission or reduces disease severity in neonates.

### 4.5. Neonatal Immune Response and ACE2 Receptor Expression in SARS-CoV-2 Infection

Neonatal immune responses to SARS-CoV-2 and the role of angiotensin-converting enzyme 2 (ACE2) receptors in susceptibility to infection have garnered significant interest. Studies have indicated that neonates possess a distinct immune profile compared to adults, characterized by an inherently lower level of pre-existing adaptive immunity and a predominance of innate immune mechanisms [67,69,70,71]. The expression of ACE2 receptors, which serve as the primary entry point for SARS-CoV-2, has been shown to be present in neonatal tissues; however, it appears to be less abundant than in adults [72]. While ACE2 is detectable in neonatal respiratory and other epithelial cells, its expression levels and the subsequent viral binding affinity are generally lower compared to older populations [73,74]. Consequently, this reduced receptor availability may contribute to a lower incidence of severe disease in neonates, though they remain susceptible to SARS-CoV-2 infection [75]. Additionally, the presence of maternally derived antibodies and the neonatal immune system’s ability to mount a response against the virus may also influence the clinical outcomes in infected neonates [76,77,78]. Understanding these differences is crucial for developing targeted preventive and therapeutic strategies to protect this vulnerable population.

## 5. Clinical Implications

The potential for transplacental transmission of SARS-CoV-2 carries several important clinical implications for both maternal and neonatal health [79,80,81]. Understanding these implications is crucial for guiding clinical management and improving the outcomes for pregnant women and their infants.

### 5.1. Maternal Health

For pregnant women, contracting COVID-19 can increase the risk of severe illness due to physiological changes in the immune system, cardiovascular system, and respiratory system during pregnancy [82,83,84]. These changes make pregnant women more susceptible to respiratory pathogens and severe pneumonia. Pregnant women with COVID-19 are at a higher risk of requiring intensive care, mechanical ventilation, and, in severe cases, experiencing mortality [85].

Moreover, COVID-19 during pregnancy can lead to complications such as preeclampsia, a condition characterized by high blood pressure and signs of damage to other organ systems, most often the liver and kidneys [43,86,87]. Pregnant women with COVID-19 are also at an increased risk of thromboembolic events due to a hypercoagulable state induced by both pregnancy and the virus [62].

### 5.2. Fetal and Neonatal Health

#### 5.2.1. Preterm Birth

Pregnant women infected with SARS-CoV-2 face a heightened risk of preterm birth [88]. This risk is linked to several factors, including the severity of maternal illness, the presence of comorbid conditions, and potential inflammatory responses triggered by the virus [89]. Preterm birth, defined as delivery before 37 weeks of gestation, carries significant risks for the neonate. Complications such as respiratory distress syndrome (RDS), which results from the underdevelopment of the lungs, are common. Preterm infants are also at risk for intraventricular hemorrhage (IVH), a type of bleeding in the brain that can lead to neurological issues. Additionally, these infants may experience long-term developmental challenges, including cognitive, motor, and behavioral problems, which necessitate ongoing medical and developmental support [90].

#### 5.2.2. Intrauterine Growth Restriction

SARS-CoV-2 infection can adversely affect placental function, potentially leading to intrauterine growth restriction (IUGR) [91,92,93]. IUGR is characterized by a fetus that fails to reach its growth potential, resulting in a lower-than-expected weight for gestational age. This condition can arise from various factors, including placental insufficiency, which impairs the delivery of essential nutrients and oxygen to the fetus. IUGR can have significant long-term implications, including increased risk for cardiovascular disease, metabolic disorders, and neurodevelopmental delays. The impact of IUGR can extend into childhood and adulthood, emphasizing the importance of monitoring and managing these pregnancies closely [94].

#### 5.2.3. Fetal Demise

Intrauterine fetal demise (IUFD), although rare, has been reported in association with maternal COVID-19 infection [95,96,97,98]. The mechanisms behind IUFD in the context of COVID-19 are not yet fully elucidated, but the proposed mechanisms include placental inflammation and thrombosis. SARS-CoV-2 infection may induce a hypercoagulable state, leading to placental vascular damage and impaired blood flow to the fetus. Additionally, the inflammatory response associated with the infection could contribute to placental dysfunction and fetal demise. These events highlight the need for the vigilant monitoring of pregnancies complicated by COVID-19 to identify and manage risks promptly [96].

#### 5.2.4. Neonatal SARS-CoV-2 Infection

Vertical transmission of SARS-CoV-2, where the virus is passed from mother to baby during pregnancy, delivery, or immediately after birth, has been documented [99,100,101]. Neonates who test positive for SARS-CoV-2 can exhibit a spectrum of symptoms, from mild respiratory issues such as cough and difficulty breathing to more severe complications like pneumonia [102]. Some infected neonates may remain asymptomatic, posing a challenge for early detection. The long-term consequences of neonatal SARS-CoV-2 infection are still under investigation, but the potential impacts include developmental delays and increased susceptibility to chronic health issues. Continuous follow-up and surveillance are essential for assessing and managing the health of these infants [103,104,105].

#### 5.2.5. Neonatal Morbidity and Mortality

Neonates born to mothers with COVID-19 may face increased morbidity and mortality due to several interconnected factors [106,107,108,109,110,111,112]. The effects of maternal illness, coupled with the risks associated with preterm birth and potential direct infection, can compromise the overall health of these infants. Neonates may require intensive care, including respiratory support and specialized monitoring, leading to prolonged hospital stays. The comprehensive care of these neonates often involves a multidisciplinary team to address both immediate needs and long-term developmental concerns. The ongoing impact of maternal COVID-19 on neonatal outcomes underscores the importance of continued research and evidence-based guidelines to improve neonatal care and outcomes [15,113].

## 6. Research on Transplacental Transmission of SARS-CoV-2

Initial reports of transplacental transmission emerged in the early stages of the pandemic, primarily through case studies and small case series [31,99,103,104]. For instance, Vivanti et al. presented one of the first documented cases of transplacental SARS-CoV-2 transmission where viral RNA was detected in the amniotic fluid and neonatal nasopharyngeal swabs, with the virus found in placental tissue as well. This case illustrated that the virus could indeed cross the placenta and result in neonatal infection [31].

Further case and case series reports followed, some of which described severe outcomes, including preterm birth, fetal distress, and neonatal complications such as respiratory distress syndrome [114,115,116,117,118,119,120,121,122,123]. These studies highlighted the need for more comprehensive research to understand the frequency, mechanisms, and consequences of transplacental transmission.

Research on the transplacental transmission of SARS-CoV-2 has produced varied results, reflecting differences in study design, patient populations, and detection methods. Some studies have detected viral RNA or proteins in placental tissues, amniotic fluid, and umbilical cord blood, providing direct evidence of potential transplacental transmission [101,124,125,126,127,128]. Additionally, cases of neonates testing positive for SARS-CoV-2 shortly after birth, with no other apparent sources of infection, suggest that vertical transmission may occur [31,103,104,129].

Conversely, other studies have not found evidence of SARS-CoV-2 in placental or fetal tissues, raising questions about the frequency and significance of transplacental transmission [60]. The variability in findings underscores the need for standardized protocols and more extensive research to determine the true risk and mechanisms of vertical transmission [118,130,131,132].

Research on the transplacental transmission of SARS-CoV-2 has produced a body of literature that is both extensive and varied [79,133,134,135,136,137]. This diversity in findings is attributable to differences in study design, population demographics, timing of sample collection, and sensitivity of detection methods. Below is an expanded examination of the research landscape, encompassing direct evidence of viral presence, indirect evidence from clinical outcomes, and the findings from placental pathology [138].

### 6.1. Detection of Viral RNA and Proteins

Several studies have reported the detection of SARS-CoV-2 RNA in placental tissues, amniotic fluid, and umbilical cord blood, suggesting that the virus can reach and potentially infect the placenta [31,124,139,140,141]. For example, SARS-CoV-2 RNA was detected in placental tissue and amniotic fluid, providing direct evidence of potential transplacental transmission [31].

Similarly, Girolamo et al. conducted a systematic review of placental pathology in cases of maternal SARS-CoV-2 infection and identified viral RNA in a significant proportion of samples, indicating that the virus can localize within the placenta [142].

### 6.2. Histopathological Findings

Histopathological examinations have revealed viral particles in syncytiotrophoblasts and other placental cells. These findings are supported by immunohistochemistry and electron microscopy studies that have visualized the virus within the placental tissue [143,144,145]. For instance, Hosier et al. (2020) documented the presence of SARS-CoV-2 in syncytiotrophoblasts using electron microscopy, corroborating molecular detection methods [140].

Histopathological examinations of placentas from mothers infected with COVID-19 have provided crucial insights into the potential mechanisms underlying transplacental transmission of SARS-CoV-2. The findings from these studies highlight several key pathological features that may contribute to or indicate the possibility of viral transmission from mother to fetus [8,13,37].

#### 6.2.1. Placental Inflammation

One of the most consistent findings across studies is the presence of increased inflammatory markers and immune cell infiltration in the placentas of COVID-19-positive mothers [8,125,146,147,148,149]. The inflammation is often characterized by chronic villitis, intervillositis, and decidual inflammation. During pregnancy, maternal immune tolerance is facilitated by an increased production of anti-inflammatory cytokines, such as interleukin-10 (IL-10), which plays a pivotal role in preventing premature labor and abortion by mitigating excessive inflammatory responses [150]. This balance is crucial as it prevents potential adverse outcomes associated with viral infections, including SARS-CoV-2.

In addition, elevated levels of pro-inflammatory cytokines such as IL-6, TNF-α, and IFN-γ have also been detected in these placental tissues. This inflammatory response can compromise the integrity of the placental barrier, which normally acts as a shield protecting the fetus from various pathogens. The disruption of this barrier might facilitate the passage of the virus from maternal to fetal circulation, thereby increasing the risk of vertical transmission. Moreover, the inflammatory environment could lead to adverse pregnancy outcomes, including preterm birth and fetal growth restriction [1,8,122].

#### 6.2.2. Vascular Changes

Significant vascular abnormalities have been documented in the placentas of COVID-19-affected pregnancies [151,152,153,154,155]. These include widespread fibrin deposition, thrombotic events, and decidual arteriopathy. Such vascular lesions can impair placental blood flow, leading to reduced oxygen and nutrient delivery to the fetus. The resulting placental dysfunction may not only jeopardize fetal health but also create a milieu conducive to viral replication and transmission. For instance, the hypoxic conditions induced by these vascular changes may alter the expression of angiotensin-converting enzyme 2 (ACE2) receptors, which are the entry points for SARS-CoV-2 into cells. This could potentially enhance the susceptibility of placental cells to viral infection [156,157].

#### 6.2.3. Placental Insufficiency

Evidence of placental insufficiency, such as decreased placental weight and abnormal placental structure, has been reported in several studies [158,159]. These changes can impair the placenta’s ability to support fetal growth and development. Mulvey et al. (2020) identified signs of placental insufficiency in their analysis of placental samples from COVID-19 positive pregnancies, highlighting the potential impact of the virus on placental function [160].

### 6.3. Indirect Evidence from Clinical Outcomes

#### 6.3.1. Neonatal Infection

Cases of neonates testing positive for SARS-CoV-2 shortly after birth provide indirect evidence of potential vertical transmission [137,161,162,163]. These cases are particularly compelling when strict infection control measures are in place, minimizing the likelihood of horizontal transmission from the environment or caregivers. Zeng et al. reported on neonates born to COVID-19 positive mothers, with some infants testing positive for the virus within hours of birth, suggesting in utero transmission [164].

#### 6.3.2. Adverse Pregnancy Outcomes

Studies have documented an association between maternal COVID-19 infection and adverse pregnancy outcomes, such as preterm birth, intrauterine growth restriction (IUGR), and fetal distress [165,166]. These outcomes could be indicative of placental dysfunction or direct fetal infection. A systematic review by Allotey et al. (2020) highlighted increased risks of preterm birth and neonatal ICU admissions among pregnant women with COVID-19, pointing to the potential impacts of the virus on pregnancy and fetal health [167].

## 7. Implications for Obstetric Care

### 7.1. Screening and Monitoring

Pregnant women should undergo routine and proactive screening for COVID-19, particularly if they present with symptoms suggestive of the virus or have been exposed to known cases. Regular testing can help identify cases early, allowing for timely intervention and reducing the risk of transmission to others [168,169]. Enhanced fetal monitoring is also crucial to ensure both maternal and fetal health are maintained. This includes frequent ultrasounds to assess fetal growth, well-being, and anatomical development. Non-stress tests should be used to evaluate fetal heart rate patterns in response to fetal movements, which can provide critical information about the baby’s health. Additionally, monitoring for potential complications such as intrauterine growth restriction or signs of distress should be an integral part of the care plan for pregnant women with COVID-19 [170].

### 7.2. Delivery Planning

The timing and mode of delivery must be tailored to the individual clinical situation, considering both the maternal and fetal factors. In cases where the maternal condition is severe or deteriorating, early delivery might be warranted to improve maternal health outcomes and mitigate risks associated with progressive illness. However, this decision must be carefully weighed against the risks associated with prematurity, including potential neonatal complications. Cesarean delivery may be considered in certain scenarios, particularly if the mother’s condition is such that a vaginal delivery could pose additional risks. Nevertheless, routine cesarean sections solely based on COVID-19 status are not generally recommended [171]. A multidisciplinary team approach, involving obstetricians, neonatologists, and infectious disease specialists, is crucial to ensure a balanced decision that prioritizes both maternal and fetal safety [170].

### 7.3. Infection Control Measures

To minimize the risk of nosocomial transmission of SARS-CoV-2 in maternity units, stringent infection control measures must be implemented. Healthcare workers should adhere to rigorous protocols, including the use of personal protective equipment (PPE) such as masks, gloves, and gowns. Adequate hand hygiene, surface disinfection, and appropriate ventilation are also essential components of an effective infection control strategy. Infected mothers should be isolated from non-infected patients to prevent cross-contamination. Implementing these measures helps protect patients, healthcare workers, and the broader hospital community, ensuring a safer environment for all [170].

### 7.4. Postpartum Care

Postpartum care should address the specific needs of mothers recovering from COVID-19 [172,173,174,175]. This includes support for breastfeeding, which may be challenging due to illness-related fatigue, respiratory issues, or stress. Healthcare providers should offer guidance and resources to assist with lactation and address any potential difficulties. Additionally, mental health support is crucial, as mothers may experience heightened anxiety, depression, or stress related to their illness and its impact on their newborn. Providing access to mental health professionals, counseling services, and peer support groups can help mitigate these challenges and promote overall well-being for both mother and baby [170].

### 7.5. Vaccination

COVID-19 vaccination is recommended for pregnant women [176]. Evidence has shown that COVID-19 vaccines are safe and effective, significantly reducing the risk of severe illness, hospitalization, and adverse outcomes related to the virus [176,177]. Vaccination not only protects the mother but also provides passive immunity to the neonate through the transfer of antibodies via the placenta. This early protection can help shield the newborn during the critical initial months of life, when they are most vulnerable to infections. Public health recommendations support vaccination as a key strategy in safeguarding maternal and neonatal health against COVID-19 [178,179].

However, while mRNA SARS-CoV-2 vaccines have been demonstrated to provide substantial initial protection, particularly in reducing severe illness and mortality, recent studies have raised concerns about waning immunity and potential immunological responses after repeated booster doses [180]. Notably, IgG4 production has been observed in individuals following multiple inoculations, which may attenuate immune responses against subsequent viral exposure [181]. Furthermore, while the transplacental transfer of maternal antibodies following vaccination has been demonstrated [182], the long-term implications of this passive immunity for infants, particularly in the context of repeated maternal vaccinations or booster doses, have not yet been comprehensively studied. As the pandemic evolves and more data become available, further research will be necessary to fully understand the long-term impact of mRNA vaccination on pediatric populations, particularly in relation to immune development and transplacental antibody transfer.

### 7.6. Long-Term Follow-Up

Infants born to mothers with COVID-19 should be closely monitored throughout their early developmental years. Long-term follow-up is essential to identify and address any potential health issues or developmental delays that may emerge. This includes regular assessments of growth, cognitive and motor development, and overall health status. Early intervention services should be considered if any abnormalities are detected. Longitudinal studies are necessary to fully understand the long-term impacts of maternal COVID-19 on offspring, including potential chronic health conditions or developmental concerns that may arise as these children grow [183,184].

## 8. Limitations and Challenges

The variability in research findings can be attributed to differences in the study design, including sample size, timing of infection during pregnancy, and methods used for detecting the virus. Some studies have reported negative results, failing to detect SARS-CoV-2 in placental or fetal tissues, which could be due to the timing of sample collection or the sensitivity of the detection methods used [84,130,132]. Indeed, the timing of maternal infection, as well as the interval between testing and viral exposure, can significantly influence the accuracy of results. For instance, while reverse transcription polymerase chain reaction (RT-PCR) is considered highly sensitive compared to other methods, it is not infallible, and false negatives can occur due to factors such as low viral load or sampling error.

There is a need for standardized protocols in studying the transplacental transmission of SARS-CoV-2. This includes consistent criteria for sample collection, processing, and analysis. Standardization would help in comparing results across studies and drawing more definitive conclusions [185].

The emergence of SARS-CoV-2 variants with differing transmissibility and virulence may influence the likelihood and mechanisms of transplacental transmission. Further research is needed to understand how these variants affect pregnancy and the risk of vertical transmission [186].

## 9. Future Research Directions

Longitudinal studies tracking pregnant women with COVID-19 and their infants are essential to understand the long-term outcomes of transplacental transmission. These studies should include follow-up assessments of child development and health [183,184].

Research focused on the molecular mechanisms of SARS-CoV-2 entry into placental cells and the subsequent immune responses is crucial [60]. Such studies can provide insights into potential therapeutic targets to prevent vertical transmission [32,187].

Investigating the efficacy of COVID-19 vaccines in preventing transplacental transmission is important. This includes assessing maternal antibody transfer to the fetus and the impact of vaccination on placental health and function [179,188].

Addressing the possibility of transplacental transmission of SARS-CoV-2 is crucial for several reasons. Firstly, understanding the risk of vertical transmission can inform guidelines for the management of pregnant women with COVID-19, including decisions regarding delivery timing and mode. Secondly, identifying potential adverse outcomes associated with transplacental transmission can help in monitoring and supporting affected neonates [189]. Lastly, understanding the mechanisms of placental infection can contribute to broader knowledge about viral pathogenesis and maternal–fetal immunity [187].

## 10. Conclusions

The transplacental transmission of SARS-CoV-2 is a complex and evolving area of study. While there is evidence suggesting that such a transmission can occur, the extent and clinical significance remain unclear. Continued research is essential to provide clearer answers and inform clinical practices to protect both pregnant women and their infants during the ongoing COVID-19 pandemic.
